# Local, state and federal face mask mandates during the COVID-19 pandemic

**DOI:** 10.1017/ice.2020.1403

**Published:** 2021-01-05

**Authors:** Judith A. Guzman-Cottrill, Anurag N. Malani, David J. Weber, Hilary Babcock, Sarah D. Haessler, Mary K. Hayden, David K. Henderson, Rekha Murthy, Clare Rock, Trevor Van Schooneveld, Sharon B. Wright, Corey Forde, Latania K. Logan

**Affiliations:** 1 Oregon Health and Science University, Portland, Oregon; 2 St. Joseph Mercy Health System, Ann Arbor, Michigan; 3 University of North Carolina, Chapel Hill, North Carolina; 4 Washington University School of Medicine, St. Louis, Missouri; 5 UMass Medical School–Baystate Campus, Springfield, Massachusetts; 6 Rush University Medical Center, Chicago, Illinois; 7 Clinical Center, National Institutes of Health, Bethesda, Maryland; 8 Cedars Sinai Health System, Los Angeles, California; 9 Johns Hopkins University School of Medicine, Baltimore, Maryland; 10 University of Nebraska Medical Center, Omaha, Nebraska; 11 Beth Israel Deaconess Medical Center, Boston, Massachusetts; 12 Queen Elizabeth Hospital, Barbados

It has been well established that severe acute respiratory coronavirus virus 2 (SARS-CoV-2), the virus that causes coronavirus disease 2019 (COVID-19), is spread primarily through respiratory droplets.^1^ In healthcare facilities with adherence to personal protective equipment (PPE) guidance, patient-to-healthcare personnel COVID-19 transmission is relatively rare. This finding demonstrates the effectiveness of face masks and respirators, in combination with eye protection, for healthcare personnel caring for patients with suspected or known COVID-19.

On April 3, 2020, the Centers for Disease Control and Prevention (CDC) released a recommendation that public citizens should wear “nonmedical cloth masks” while in public places to help prevent the spread of SARS-CoV-2.^2^ The initial rationale behind this recommendation was for “source control,” to limit the emission of virus-containing respiratory droplets from infected people during their contagious period. As the viral transmission dynamics were better understood, another pandemic challenge was realized: up to 40% of infected people are asymptomatic but can shed high levels of SARS-CoV-2 from their respiratory tracts, and they contribute to more than half of viral transmissions.^3–6^ Thus, the universal use of face masks for source control among the general public became a top priority in the pandemic mitigation strategy.

Despite the evidence supporting universal public face masks, guidance varies widely from state to state. As of November 28, 2020, the US governors of 35 states have issued emergency orders for statewide mandatory mask requirements.^7^ In the states lacking statewide mandates, some local leaders within those states have issued face mask requirements for their cities or counties. Across the United States, variations regarding face mask exceptions add even more complexity. Examples include exempting children <5 years of age in Oregon,^8^ compared to children <10 years of age in Texas.^9^ Additionally, many states do not require face masks in places of worship and other specific settings.^9–11^

The Society of Healthcare Epidemiology of America (SHEA) supports and recommends face-mask mandates as an effective strategy to decrease SARS-CoV-2 community transmission. Mask have been proven to decrease person-to-person respiratory viral transmission and to decrease community COVID-19 rates. These decreases will reduce the pressures on healthcare facilities as the number of COVID-19–related hospitalizations continue to surge across the country and ultimately, masks will save lives.

Several studies have demonstrated that face masks decrease the spread of SARS-CoV-2 virus. The primary objective is to provide “source control” of infected individuals, and face covering blocks a large proportion of the person’s respiratory secretions during talking, deep exhalation, singing, and similar actions. Face masks can decrease the number of expelled large droplets and aerosols, the concentration of virus-laden droplets, and the distance that secretions travel from the source person.^6^ As a result, the exposure risk to other people is diminished.

Although cloth face masks are not equivalent to medical-grade PPE used in healthcare settings, recent studies demonstrate that cloth face masks do provide some protection to individuals exposed to contagious persons in the community. Depending on the material used, cloth masks can provide filtration of droplets and particles <10 µm, can utilize static electricity to capture particles, or may help repel moisture.^6^

The safety of cloth face masks has been questioned, including unsubstantiated claims that face masks can cause hypoxia. To date, no national medical society has identified any true risk that outweighs the benefits of wearing face masks in public during the COVID-19 pandemic. In addition, 3-layer masks are not associated with a decline in oxygen saturation in older persons, and they may be protective for children with exercise-induced asthma.^12,13^ The American Lung Association recommends public face masks for people with chronic lung disease in addition to the other public health–recommended preventive strategies that all should follow: maintaining physical distance from others, avoiding crowded areas, and performing frequent hand hygiene.^14^

Across the United States, community-level COVID-19 rates vary dramatically. Notably, public mask requirements appear to correlate with lower COVID-19 rates. In July 2020, the governor of Kansas issued an executive order requiring public face masks, but county officials had the authority to opt out of the mandate.^16^ In the 24 counties that accepted the face mask requirement, the COVID-19 incidence decreased.^17^ Simultaneously, the COVID-19 incidence dramatically increased in the counties that opted out of the mask mandate.^17^ Similar trends comparing counties with and without public face mask requirements have also been reported in Missouri.^18^

Many hospitals and healthcare systems are currently operating at crisis capacity, and their inpatient surge capacity plans are not meeting the widening gap. Temporary “overflow facilities” are being erected, adults are overflowing into children’s hospitals, and hospital staffing shortages are not being met. Facilities continue to face challenges in unreliable PPE supply chains, and commercial laboratory turnaround times are again lengthening due to dramatic increases in patient testing. Until a vaccine is widely available, the only effective way to reverse this dire situation is to “flatten the curve” and for the general public to do everything possible to decrease viral transmission. Public face mask mandates are a critical part of this strategy. As CDC Director Dr Robert Redfield stated, “Cloth face coverings are one of the most powerful weapons we have to slow and stop the spread of the virus—particularly when used universally within a community setting. All Americans have a responsibility to protect themselves, their families, and their communities.”^19^

We are facing unfortunate challenges during this pandemic, including a growing distrust in public health agencies and the politicization of wearing face masks in public. Across the country, some citizens hold the opinion that wearing a face mask is taking away their individual freedom or civil liberty. However, widespread mask requirements should be regarded through a very different lens. SARS-CoV-2 is a respiratory virus and wearing a face mask will decrease person-to-person spread.^6,20^ Mask mandates that include minimal exceptions will lead to a reduction in community COVID-19 rates, decrease hospitalizations, and save lives. Reducing community prevalence and transmission will allow us to find our individual and community freedoms again: freedom to travel, send students back to classrooms, return to indoor dining, and safely reopen the economy. Most importantly, mask mandates will decrease the enormous pressures placed on healthcare facilities and decrease the occupational risks that our frontline healthcare workers are currently facing while working in crisis capacity settings.
